# Comparative effectiveness of two popular weight loss programs in women IV: quality of life and diet satisfaction

**DOI:** 10.1186/1550-2783-8-S1-P3

**Published:** 2011-11-07

**Authors:** Andrew Jagim, Michelle Mardock, Brittanie Lockard, Jonathon Oliver, Mike Byrd, Sunday Simbo, Julie Kresta, Claire Baetge, Peter Jung, Majid Koozehchian, Deepesch Khanna, Mike Greenwood, Chris Rasmussen, Richard Kreider

**Affiliations:** 1 Exercise & Sport Nutrition Lab. Texas A&M University, College Station, TX 77843, USA

## Background

A number of commercial diet and exercise programs are promoted to help people lose weight and improve fitness. However, few studies have compared the effects of following different types of exercise and diet interventions on weight loss, health, and quality of life. The purpose of this study was to compare the efficacy of a more structured meal plan based diet intervention and supervised exercise program that included resistance-exercise to a traditional point based diet program with weekly counseling and encouragement to exercise.

## Methods

Fifty-one sedentary women (35±8 yrs, 163±7 cm; 90±14 kg; 47±7% body fat, 34±5 kg/m^2^) were randomized to participate in the Curves (C) or Weight Watchers (W) weight loss programs for 16-wks. Participants in the C program were instructed to follow a 1,200 kcal/d diet for 1-week, 1,500 kcal/d diet for 3 weeks, and 2,000 kcals/d diet for 2-weeks consisting of 30% carbohydrate, 45% protein, and 30% fat. Subjects then repeated this diet. Participants also participated in the Curves circuit resistance training program 3 days/week for 30-minutes. This program involved performing 30-60 seconds of bi-directional hydraulic-based resistance-exercise on 13 machines interspersed with 30-60 seconds of low-impact callisthenic or Zumba dance exercise. Participants in the W group followed the W point-based diet program, received weekly counseling, and were encouraged to increase physical activity. Eating satisfaction and SF-36 quality of life and questionnaires were obtained at 0, 4, 10, & 16 wks and analyzed by multivariate analysis of variance (MANOVA) with repeated measures. Data are presented as changes from baseline for the C and W groups, respectively.

## Results

MANOVA analysis of SF36 quality of life indices revealed an overall Wilks’ Lamda time effect (p=0.09) with no significant diet (p=0.44) or time x diet effect (p=0.45).Within subjects univariate analysis revealed that both programsincreased rating of physical function (17.3±36%, p=0.002), role physical (17.5±56%, p=0.03), role emotional (11.8±30 %, p=0.02), vitality(20.8±35%, p=0.001), role emotion (19.1±30 %, p=0.001), bodily pain (19.1±34 %, p=0.001) and general health (12.6±23 %, p=0.001) with no time effect on social functioning (3.0±20 %, p=0.57) following 16 weeks. No significant interactions were seen between diet groups. MANOVA analysis of eating satisfaction inventories revealed significant within subjects time effects (p=0.001) with a trend toward a significant interaction effect (p=0.059). Univariate analysis revealed that both programs decreased rating of appetite (-0.5±1.5, p=0.003), amount of energy (-1.6±2.0, p=0.001), and overall quality of diet (-2.5±2.7, p=0.001) with no time effect on hunger (0.1±1.6, p=0.38) or satisfaction from food (-0.3±2.0, p=0.64) following 16 weeks. Perceptions of feelings of fullness were significantly higher in the C group (C 0.4±1.9, 0.0±1.7, 0.5±1.4; W -0.8±1.8,-0.7±1.9, -0.8±1.4; p=0.04).

## Conclusion

Results indicate that participation in the C and W programs generally improve markers of quality of life and participants following the C program experience fullness to a greater fullness than those following the W program.

## Funding

Supported by Curves International (Waco, TX)

